# A Novel Feature Selection Technique for Text Classification Using Naïve Bayes

**DOI:** 10.1155/2014/717092

**Published:** 2014-10-28

**Authors:** Subhajit Dey Sarkar, Saptarsi Goswami, Aman Agarwal, Javed Aktar

**Affiliations:** Department of Computer Science and Engineering, Institute of Engineering and Management, West Bengal 700091, India

## Abstract

With the proliferation of unstructured data, text classification or text categorization has found many applications in topic classification, sentiment analysis, authorship identification, spam detection, and so on. There are many classification algorithms available. Naïve Bayes remains one of the oldest and most popular classifiers. On one hand, implementation of naïve Bayes is simple and, on the other hand, this also requires fewer amounts of training data. From the literature review, it is found that naïve Bayes performs poorly compared to other classifiers in text classification. As a result, this makes the naïve Bayes classifier unusable in spite of the simplicity and intuitiveness of the model. In this paper, we propose a two-step feature selection method based on firstly a univariate feature selection and then feature clustering, where we use the univariate feature selection method to reduce the search space and then apply clustering to select relatively independent feature sets. We demonstrate the effectiveness of our method by a thorough evaluation and comparison over 13 datasets. The performance improvement thus achieved makes naïve Bayes comparable or superior to other classifiers. The proposed algorithm is shown to outperform other traditional methods like greedy search based wrapper or CFS.

## 1. Introduction

The proportion of unstructured data to structured data has been rising consistently in the last few decades [1, 2]. To extract meaningful information from these large corpora of text data, both statistical/machine learning techniques and linguistic techniques are applied. Text classification has various applications in the form of email classification, sentiment analysis, language identification, authorship identification, influential blogger detection, and topic classification to name a few. Classification is called supervised learning as it requires training data. The classification algorithm builds the necessary knowledge base from training data and then a new instance is classified in predefined categories based on this knowledge. As an example of a classification task, we may have data available on various characteristics of breast tumors where the tumors are classified as either benign or malignant. Now, given an unlabeled tumor, the classifier will map it as either benign or malignant. The classifier can be thought as a function which maps an instance or an observation based on the attribute values to one of the predefined categories. Text classification is a part of classification, where the input is texts in terms of documents, emails, tweets, blogs, and so forth. One of the problems with text classification is much higher input size. More formally, given a set of document vectors {*d*_1_; *d*_2_; …; *d*_*n*_} and their associated class labels *c*(*di*) ∈ {*c*_1_; *c*_2_; …; *c*_*l*_}, text classification is the problem of assigning the class label to an unlabeled document *d*.

Most classification algorithms require sufficient training data, which adds to the space complexity as well as increased training time. So the capability of a classifier to give good performance on relatively less training data is very critical. Naïve Bayes classifier is one such classifier which scores over the other classifiers in this respect. Naïve Bayes model is the simplest of all the classifiers in the way that it assumes that all the attributes are independent of each other in the context of the class [3–7]. It is mentioned in [8] that naïve Bayes is widely used because of its simplicity, though it is one of the classifiers noted to have poor accuracy in tasks like text categorization. The unique contributions of the paper are as follows.

(i) We offer a simple and novel feature selection technique for improving naïve Bayes classifier for text classification, which makes it competitive with other standard classifiers.
(ii) Contrary to conventional feature selection methods, we employ feature clustering, which has a much lesser computational complexity, and equally if not more effective outcome, a detailed comparison has been done.
(iii) Our approach employs the below steps:
  (a) Step  1: chi-squared metric is used to select important words;
  (b) Step  2: the selected words are represented by their occurrence in various documents (simply by taking a transpose of the term document matrix);
  (c) Step  3: a simple clustering algorithm like *K*-means is applied to prune the feature space further, in contrast to conventional methods like search and one word/feature corresponding to each cluster that is selected.
(iv) The superiority of our performance improvement has been shown to be statistically significant.

The organization of the paper is as follows. In Section 2, the theoretical foundation of naïve Bayes classifier is discussed. In Section 3, a brief overview of feature selection is provided. In Section 4, we present our algorithm with necessary illustration. In Section 5, we discuss experimental setup and in Section 6 the results of various studies and their analysis are presented.  Section 7 contains the conclusion and future scope of work.

## 2. Naïve Bayes Classifier and Text Classification

Naïve Bayes is based on conditional probability, and following from Bayes theorem, for a document *d* and a class *c*, it is given as

(1) $$\begin{array}{c} P\left(c\;|\;d\right)=\frac{P\left(d\;|\;c\right)P\left(c\right)}{P\left(d\right)}. \end{array}$$

The most likely class (maximum a posteriori) is given by

(2) $$\begin{array}{c} c_{\text{MAP}}=\underset{c\in C}{\arg\;\max}\;P\left(cd\right), \end{array}$$

(3) $$\begin{array}{c} \alpha \;\underset{c\in C}{\arg\;\max}\;P\left(dc\right)P\left(c\right), \end{array}$$

(4) $$\begin{array}{c} \alpha \;\underset{c\in C}{\arg\;\max}\;P\left(t_{1},t_{2},\ldots ,t_{n}\;|\;c\right)P\left(c\right), \end{array}$$

where the document *d* is represented by different features like *t*_1_, *t*_2_,…, *t*_*n*_, respectively. (Typically the features correspond to words.) The naïve Bayes assumptions depict all features is independent of each other. This assumption transforms (4) as follows:

(5) $$\begin{array}{c} \alpha \;\underset{c\in C}{\arg\;\max}\;P\left(c_{j}\right)\underset{t\in T}{\prod }P\left(t\;|\;c\right). \end{array}$$

In a previous work of the authors, naïve Bayes has been compared with few other popular classifiers like support vector machine (SVM), decision tree, and *k* nearest neighbor (kNN) on various text classification datasets [9].  Table 1 summarizes the findings. Naïve Bayes's performance was the worst among the classifiers.

We argue that the reason for this lesser accurate performance is the assumption that all features are independent. The authors carry out extensive empirical analysis of feature selection for text classification and observe SVM to be the superior classifier [10], which indirectly supports our claim of naïve Bayes's poor performance.

One of the popular methods to represent a document is by using a bag of words (BoW) or vector space model using term document matrix, where each document is represented by the words present in the document after some preliminary transformations, rather than raw counts (order of the word is ignored). One such weighting scheme uses both the term frequency and the inverse document frequency given by (tf-idf) [13], which balances the number of occurrences of a word in a particular document and novelty of that word:

(6) $$\begin{array}{c} w_{t,d}=\log\left(1+\text{t}{\text{f}}_{t,d}\right)\times \text{lo}{\text{g}}_{10}\left(\frac{N}{\text{d}{\text{f}}_{t}}\right). \end{array}$$

So each word represents the features of documents and the weights described by (6) are the values of the feature, respectively, for that particular document. Using our proposed method, we want to modify (5) as follows:

(7) $$\begin{array}{c} \underset{c\in C}{\arg\;\max}\;P\left(c_{j}\right)\underset{t\in A}{\prod }P\left(t\;|\;c\right), \end{array}$$

where *n*(*A*) ≪ *n*(*F*), selecting *A* in a manner such that they are less dependent on each other.

A survey on improving Bayesian classifiers [14] lists down (a) feature selection, (b) structure extension, (c) local learning, and (d) data expansion as the four principal methods for improving naïve Bayes. We focus on* feature selection* in our proposition. The attribute independence assumption can be overcome if we use Bayesian network; however, learning of an optimal Bayesian network is an NP hard problem [15].

In [16], the authors have proposed an improvement for naïve Bayes classification using a method name as auxiliary feature method. The idea is to find an auxiliary feature to each independent feature such that the auxiliary feature increases separability of the class probabilities than the current feature. As we need to determine the auxiliary feature for all features, this method has high computational complexity. In [8], the authors propose a novel method of improving the naïve Bayes by multiplying each conditional probability with a factor, which can be represented by chi-squared or mutual information. Reference [17] proposes a word distribution based clustering based on mutual information, which weighs the conditional probabilities based on the mutual information content of the particular word, based on the class. Our proposed method will have an advantage as in the first step we reduce the feature sets using a simple univariate filter before applying clustering.

## 3. Feature Selection

Feature selection is one of the most important data preprocessing steps in data mining and knowledge engineering. Let us say we are interested in a task (*T*), which is finding employees prone to attritions. Each employee is represented by various attributes/features (*Fn*) like their age, designation, marital status, average working hours, average number of leaves taken, take-home salary, last ratings, last increments, number of awards received, number of hours spent in training time from the last promotion, and so forth. In feature selection, the idea is to select best few features (*Fm*) from the above, so as to we perform equivalently in performing the task (*T*), in terms of some evaluation measure (*M*). (Generally *m* ≪ *n*.) So for a classification task, a standard evaluation measure like classification accuracy and *F*-Score, and so forth, and for clustering it can be internal measures like silhouette width or an external measure like purity.

Feature selection offers the following three advantages:

- *better model understandability and visualization*: it might not be possible to reduce to a two-dimensional or a three-dimensional feature set, but even if we want to visualize with a combination of two or three features, the combinations will be much lesser in the reduced feature space;
- *generalization of the model and reduction over fitting*: as a result better learning accuracy is achieved;
- * efficiency in terms of time and space complexity*: for both training and execution time.

Feature selection approaches can be broadly classified as filter, wrapper, and embedded.

*Filter Approach*. This is the most generic of all the approaches and works irrespective of the data mining algorithm that is being used. It typically employs measures like correlation, entropy, mutual information, and so forth which analyzes general characteristic of the data to select an optimal feature set. This is much simpler and faster to build compared to embedded and wrapper approaches; as a result, this method is more popular to both academicians and industry practitioner. However, it is to be noted that wrapper and embedded methods often outperform filter in real data scenarios. 

*Embedded Approach*. Feature selection is a part of the objective function of the algorithm itself. Examples of the same are decision tree, LASSO, LARS, 1-norm support vector, and so forth. 

*Wrapper Approach*. In this method, the wrapper is built considering the data mining algorithm as a black box. All combinations of the feature sets are used and tested exhaustively for the target data mining algorithm and it typically uses a measure like classification accuracy to select the best feature set. Because of the “brute force” approach, these methods tend to be computationally extensive.

In terms of outputs, it can be set of ranked features or optimal subset of features. We can classify the approaches as either univariate or multivariate. In the univariate class, all features are treated individually and ranked (some of the popular metrics are information gain, chi-square, and Pearson correlation coefficient).

Correlation feature selection (CFS) is a very popular example of such multivariate techniques [18]:

(8) $$\begin{array}{c} r_{zc}=\frac{k{\overset{-}{r}}_{zi}}{\sqrt{k+k*\left(k-1\right)*{\overset{-}{r}}_{ii}}}, \end{array}$$

where *r*_*zc*_ indicates worth of features subset. *r*_*zc*_ is the average of correlation between the features and the target variable. *r*_*ii*_ is the average intercorrelation between the components.

The one with the highest *r*_*zc*_ is selected.

Both filter and wrapper methods can employ various search strategies. As exhaustive search is computationally complex, various other variants like greedy (both sequential backward and forward), genetic search, hill climbing, and so forth are used for better computational efficiency. Our proposed algorithm based on feature clustering provides better computation complexity because of the following reasons.

1. It does not follow the wrapper method, so that many numbers of combinations do not need to be enumerated.
2. We employ clustering, which is not as involved as search [19, 20].
3. We effectively consider both the univariate and multivariate nature of the data.
4. There is no additional computation required as the term document matrix is invariably required for most of the text classification tasks.

Reference [20] proposes a clustering based feature selection, and we would like to highlight the following differences with the method we have proposed. Firstly, we employ a partition based clustering instead of a hierarchical one; secondly, even in case of clustering, we limit ourselves to a much pruned dataset as in the first step we are retaining only the most relevant ones. The authors use maximal information compression index (MICI) as defined in [19] to measure the similarity of the features which is also an additional computational step. For finding the prototype feature, average distance from all the features in the cluster is taken, where other simpler versions could have been applied.

In [19], the authors define a measure of linear dependency, maximal information compression index (*λ*2) as the smallest eigenvalue of Σ, and the value of *λ*2 is zero when the features are linearly dependent and increases as the amount of dependency decreases:

(9) Maximal  Information  Compression  Index (λ2)  =minimum(Λxy),

where Λ is a (*n*∗*n*) matrix, where *n* is number of features and each of diagonal entries holds the corresponding eigen values:

(10) $$\begin{array}{c} \Lambda =\left[\begin{array}{ccc} \lambda_{1} & \cdots & \\ \vdots & \ddots & \vdots \\ & \cdots & \lambda_{n} \end{array}\right]. \end{array}$$

We have also added an empirical comparison between FS-CHICLUST and wrapper with greedy search and multivariate filter search using CFS in Table 9, in Section 6.

## 4. Our Proposition

Naïve Bayes is one of the simplest and hence one of the most widely used classifiers. However, this often does not produce results comparable with other classifiers because of the “naïve” assumption; that is, attributes are independent of each other. Performance of naïve Bayes further deteriorates in the text classification domain, because of the higher number of features. Our proposed method works on the term document matrix [13]. We firstly select the important words based on the chi-squared value, that is, selecting only those words which have a value higher than a threshold. We have taken this as “0” in our experimental study. The chi-squared statistics is detailed below.

### 4.1. Chi-Squared (*χ* ^2^ Statistic)

Chi-squared is generally used to measure the lack of independence between *t* and *c* (where *t* is for term and *c* is for class or category) and compared to the *χ* ^2^ distribution with one degree of freedom. The expression for *χ* ^2^ static is defined as

(11) $$\begin{array}{c} \chi_{\left(t,c\right)}^{2}=\frac{D\times {\left(PN-MQ\right)}^{2}}{\left(P+M\right)\times \left(Q+N\right)\times \left(P+Q\right)\times \left(M+N\right)}, \end{array}$$

where *D* is the total number of documents. *P* is the number of documents of class *c* containing term *t*. *Q* is the number of documents containing *t* occurring without *c*. *M* is the number of documents class *c* occurring without *t*. *N* is the number of documents of other classes without *t*.

Next, we take the selected words and represent them by their occurrence in the term document matrix. So, if there are three documents *d*1, *d*2, and *d*3 and there are four words *w*1, *w*2, *w*3, and *w*4, respectively, then the term document matrix is represented as shown in Table 2.

Then we argue that the individual features, that is, the words, can be represented as their occurrence in the documents so *w*1 can be represented as a vector {1.1,2.3,1.1} and if by this representation two words have a smaller distance between them, then that means they are similar to each other. The weighing scheme is tf-idf as explained in Section 2.

Finally, we have applied *K*-means clustering, which is one of the simplest and most popular clustering algorithms. One of the inputs *K*-means expects is the value of *K*, that is, the number of clusters.

The optimal number of clusters is one of the open questions in clustering [21]. For our present setup, we start with the square root of *n* (of the reduced set of step 1, using chi-squared) as per [22] and proceed up to *n*/2. As indicated in [20], a feature clustering method may need a few iterations to come to an optimal or near optimal number of features but this is much lesser than a search based approach using a filter or wrapper method.

The algorithm is described below; the algorithm accepts three parameters:

(a) the term document matrix corresponding to the text corpora: *TM*;
(b) number of clusters (starting point can be square root of *n*): nc,
(c) threshold takes a float as input: thresh.

Thresh is taken as “0” in the current case, and this can also be used by determining the 10th percentile or so on. It produces the reduced feature set as the output.

*Algorithm FS-CHICLUST*.

Input: Term Document Matrix *TM* (*D* × *W*) dimension *D* indicates No. of documents and *W* indicates no. of words an entry *TM*_*ij*_ indicates the corresponding tf-idf

No. of Clusters (nc), 

Thresh (Default Value 0)

Output: Feature Set *F* initially empty set {}

**Step 1 .**

We apply the feature selection technique based on chi-squared on the entire term document matrix to compute chi-squared (CH) value corresponding to each word.

**Step 2 .**

We select only those words that have a CH value greater than* thresh.*

**Step 3 .**

We form a new term document matrix (*M*) which consists of only those important words as selected in Step 2.

**Step 4 .**

We transpose this new term document matrix (*M*) and each row represents a word. The transposed matrix is denoted by “*N*.”

**Step 5 .**

We create “nc” clusters on “*N*.”

**Step 6 .**

We select the most representative words from each cluster, which is the closest to the clustering centre and add them one by one to *F* such *n*(*F*) = nc.

**Step 7 .**

The Euclidian norm is calculated for each point in a cluster, between the point and the center. The one nearest to the center is selected.

## 5. Experimental Setup

This section describes details about the setup of the experiment. It covers details about the datasets that are used and different preprocessing techniques that were applied. The software tool and packaged that are used, Hardware and software details of the machine, on which the experiment was carried out.

### 5.1. Dataset Information

The detailed information of the datasets used in our experimental setup has been summarized in Table 3.

### 5.2. Methodology

The basic steps followed for the experiment are described below for reproducibility of the results.

- Text documents are stripped of space and punctuation.
- Numbers and stop words are removed.
- Stemming and lowercasing are applied.
- The term document matrix is prepared on the processed document. The weighing scheme that has been used is the tf-idf.
- The term document matrix is split into two subsets, 70% of the term document matrix is used for training, and the rest 30% is used for testing classification accuracy [22].
- The so-produced term document matrix is used for our experimental study.
- We compare the results with other standard classifiers like decision tree (DT) SVM and kNN.
- We also compare execution time taken by FSCHICLUST with other approaches like wrapper with greedy search and multivariate filter based search technique based on CFS.

The hardware and software used are as follows:

 processor: Intel Core Duo CPU T6400 @ 2.00 GHZ;
 RAM: 4 GB;
 OS: Windows 7 Ultimate SP1;
 R: version 2.15.3 [23].

Various standard R packages used are in [24–26], respectively.

## 6. Results and Analysis

### 6.1. Results

We have used classification accuracy, which is a measure of how well a document is classified into its appropriate class. It is simply the % of* # Correctly Classified Documents/# Total Documents*. All the classification accuracies have been computed on testing dataset. We present the following evaluation and comparison, respectively.

**Figure 1:**
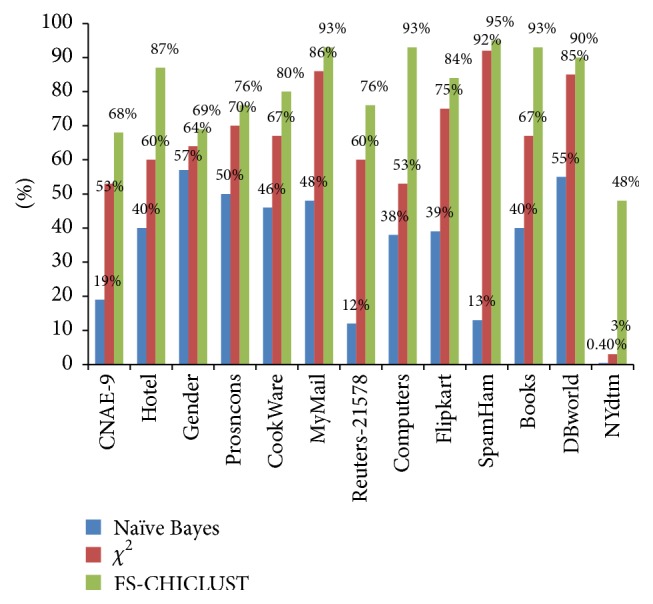
Improvement FS-CHICLUST.

- Classification accuracy on the test dataset using (a) naïve Bayes, (b) chi-squared with naïve Bayes, and (c) FS-CHICLUT with naïve Bayes is computed. The result is summarized in Table 4 and Figure 1.
- Using FS-CHICLUST, we can significantly reduce the feature space. The total number of features and reduced number of features using (a) chi-squared and (b) FSCHICLUST are displayed in Table 5.

**Figure 2:**
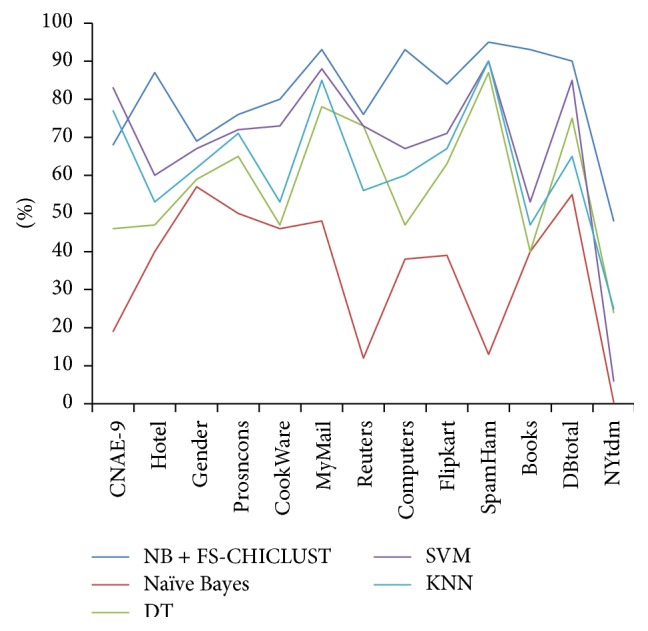
FS-CHICLUST with other classifiers.

In Table 6, we summarize % reduction of feature set and the % improvement of classification accuracy over all the datasets between simple naïve Bayes and FS-CHICLUST with naïve Bayes.

(iii) We compare the results of FSCHICLUT and naïve Bayes with other classifiers like kNN and SVM and decision tree (DT), which makes naïve Bayes (NB) comparable with other classifiers, the results are summarized in Table 7, and the classifier accuracy is also displayed in line chart in Figure 2.

We employed Friedman's nonparametric test to compare the results of the classifiers. Other popular measures like ANOVA could have been used. Friedman test has been given preference because of no assumption about the underlying model.

Given data, [*x*_*ij*_]_*n*∗*k*_ is to be replaced by [*r*_*ij*_]_*n*∗*k*_, where *r*_*ij*_ denotes the ranks, respectively; in case of a tie, *r*_*ij*_ is replaced by an average value of the tied ranks:

(12) $$\begin{array}{c} r_{.j}=\frac{1}{n}\overset{n}{\underset{i=1}{\sum }}r_{ij}. \end{array}$$

Comparing mean ranks, we see that our method has a better mean rank than the other four methods, and the mean ranks for all the methods are summarized in Table 8.

Below are the details of the Friedman rank sum test:

(13) $$\begin{array}{c} \text{Friedman}\;\text{chi-squared}=39.9447,\\ \text{df}=4,\;\;P\text{ value}=4.444e-08. \end{array}$$

The *P* value is very less, so the null hypothesis that the difference in ranks is not significant is rejected and we can conclude that FSCHICLUST has significantly better performance than other classifiers.

(iv) We compare the execution time of FSCHICLUST with other approaches like
  - wrapper with greedy search (forward),
  - multivariate filter using CFS (using the best first search).

The results are shown in Tables 9(a) and 9(b), respectively.

### 6.2. Analysis

#### 6.2.1. Increase in Accuracy

(1) Using FS-CHICLUST, we can achieve significant improvement over naïve Bayes. Student's paired *t*-test is performed on the 11 datasets (degree of freedom 10) that we used in our experiment. NULL hypothesis is rejected if *P* < 0.01 (Table 4):

(14) $$\begin{array}{c} t=-9.5458,\;\;\text{df}=12,\\ P\text{ value}=5.904e-07. \end{array}$$

So the difference is indeed significant.

#### 6.2.2. Considerable Reduction in Feature Space

On one hand, we have significant improvement in terms of classification accuracy; on the other hand, we could reduce the number of features from univariate chi-square. What we observe is that at significance level of 0.05 there is a significant reduction in our proposed method, compared to reduction achieved through chi-square alone (Table 5):

(15) $$\begin{array}{c} t=2.517,\;\;\text{df}=12,\\ P\text{ value}=0.02706. \end{array}$$

#### 6.2.3. Comparison with Other Classifiers

FSCHICLUST makes naïve Bayes competitive with other classifiers; in fact, the average rank is the lowest among the classifiers (Table 8), and the nonparametric Friedman rank sum test corroborates the statistical significance.

#### 6.2.4. Comparison with Other Feature Selection Methods

We have compared the execution time and classification accuracy with greedy forward search based wrapper method (Table 9(a)) and CFS based multivariate filter method which employs the best first search (Table 9(b)). Our proposed method has got much better result both on execution time and on classification accuracy.

In Table 10, there are *P* values corresponding to the comparison with greedy based wrapper search and CFS. This shows that there is a significant difference between the two results.

## 7. Conclusion

Our previous study and works of other authors show naïve Bayes to be an inferior classifier especially for text classification. We have proposed a novel two-step feature selection algorithm which can be used in conjunction with naïve Bayes to improve the performance. We have evaluated our algorithm FS-CHICLUST over thirteen datasets and did extensive comparisons with other classifiers and also with other feature selection methods like greedy based wrapper, CFS, and so forth. Below is the summary of our findings.

- FS-CHICLUST is successful in improving the performance of naïve Bayes. The improvement in performance is statistically significant.
- FS-CHICLUST not only improves performance but also achieves the same with further reduced feature set. The reduction compared to univariate chi-square is statistically significant.
- Naïve Bayes combined with FS-CHICLUST gives superior performance than other standard classifiers like SVM, decision tree, and kNN.
- Naïve Bayes combined with FS-CHICLUST gives better classification accuracy and takes lesser execution time than other standard methods like greedy search based wrapper and CFS based filter approach.

So FS-CHICLUST will improve naïve Bayes's performance for text classification and make this simple to implement intuitive classifier suitable for the task of text classification.

We have used *K*-means clustering which is the simplest among the clustering algorithms have been applied here for feature clustering; we can extend this work by employing other advanced clustering techniques. The work can be extended to further limit choice of no. of clusters and use other text presentation schemes like topic clustering.

## Figures and Tables

**Table 1:** Comparison of classifiers based on classification accuracy.

Classification algorithms	CNAE-9	SPAMHAM	Hotel dataset
Decision tree	46%	87%	47%
SVM	83%	90%	60%
Naive Bayes	19.8%	13%	40%
k-NN	77%	90%	53%

**Table 2:** Term document matrix example.

Document	*w*1	*w*2	*w*3	*w*4
*d*1	1.1	0	2	3
*d*2	2.3	0	0	.5
*d*3	1.1	0	0	.25

**Table 3:** Characteristics of the datasets.

Datasets	Number of documents	Number of terms	Number of classes/categories
CNAE-9	1080	856	9
Hotel	50	3360	2
Gender	3232	100	2
Prosncons	2000	1493	2
CookWare	50	2370	2
MyMail	194	4466	2
Reuters^*^	279	3170	3
Computers	50	3358	2
Flipkart	400	3043	2
SpamHam	5572	6631	2
Books	50	3300	2
DBWorld	64	3723	2
NYdtm	3104	5587	27

The datasets can be mostly found at [11, 12].

^*^Data for three classes have been used for Reuters.

**Table 4:** Classification accuracy rate of naïve Bayes during the three phases of experiment.

Datasets	Naïve Bayes	Chi-squared	FS-CHICLUST
CNAE-9	19%	53%	**68%**
Hotel	40%	60%	**87%**
Gender	57%	64%	**69%**
Prosncons	50%	70%	**76%**
CookWare	46%	67%	**80%**
MyMail	48%	86%	**93%**
Reuters-21578	12%	60%	**76%**
Computers	38%	53%	**93%**
Flipkart	39%	75%	**84%**
SpamHam	13%	92%	**95%**
Books	40%	67%	**93%**
DBWorld	55%	85%	**90%**
NYdtm	.4%	3%	**48%**

**Table 5:** Feature reduction of naïve Bayes after the three phases of experiment.

Datasets	Total features	Features using chi-square	Using FS-CHICLUST
CNAE-9	856	80	32
Hotel	3360	6	3
Gender	100	26	12
Prosncons	1493	23	11
CookWare	2370	6	3
MyMail	4466	25	14
Reuters-21578	3170	38	11
Computers	3358	8	4
Flipkart	3043	25	14
SpamHam	6631	150	30
Books	3300	4	2
DBWorld	3723	5	2
NYdtm	5587	44	6

**Table 6:** Summary of feature reduction and classification accuracy improvement.

Datasets	% reduction	% improvement in classification accuracy
CNAE-9	96.3%	258%
Hotel	99.9%	118%
Gender	88%	21%
Prosncons	99.3%	52%
CookWare	99.9%	74%
MyMail	99.7%	94%
Reuters-21578	99.7%	533%
Computers	99.9%	145%
Flipkart	99.5%	115%
SpamHam	99.5%	631%
Books	99.9%	133%
DBWorld	99.9%	66%
NYdtm	99.2%	1000%

**Table 7:** Comparison of proposed method with other classifiers.

Datasets	NB + FS-CHICLUST	Naïve Bayes	DT	SVM	kNN
CNAE-9	**68%**	19%	46%	83%	77%
Hotel	**87%**	40%	47%	60%	53%
Gender	**69%**	57%	59%	67%	62%
Prosncons	**76%**	50%	65%	72%	71%
CookWare	**80%**	46%	47%	73%	53%
MyMail	**93%**	48%	78%	88%	85%
Reuters	**76%**	12%	73%	73%	56%
Computers	**93%**	38%	47%	67%	60%
Flipkart	**84%**	39%	63%	71%	67%
SpamHam	**95%**	13%	87%	90%	90%
Books	**93%**	40%	40%	53%	47%
DBTotal	**90%**	55%	75%	85%	65%
NYtdm	**48%**	.4%	24%	6%	25%

**Table 8:** Comparison of proposed method with other classifiers.

Algorithms	Mean rank
FS-CHICLUST	1.18
SVM	2
kNN	2.77
DT	3.91
NB	4.95

**(a):** 

Datasets	Wrapper forward search greedy		NB + FS-CHICLUST	
	Execution time	Classification accuracy	Execution time	Classification accuracy
CNAE-9	53.76 min	54%	0.81 min	68%
Hotel	44.89 min	60%	3.92 min	87%
Gender	3.10 min	59%	0.06 min	69%
Prosncons	71.82 min	51%	1.70 min	76%
CookWare	48.19 min	53%	3 min	80%
MyMail	120.41 min	85%	5.10 min	93%
Reuters	119.30 min	19%	3.80 min	76%
Computers	65.75 min	40%	4.25 min	93%
Flipkart	105.48 min	67%	4.12 min	84%
SpamHam	191.41 min	14%	5.15 min	95%
Books	42 min	33%	3.85 min	93%
DBTotal	50.52 min	60%	8.1 min	90%

**(b):** 

Datasets	Filter (CFS)		NB + FS-CHICLUST	
	Execution time	Classification accuracy	Execution time	Classification accuracy
CNAE-9	85.80 min	68%	0.81 min	68%
Gender	22.02 min	55%	0.06 min	69%
Prosncons	293.40 min	70%	1.70 min	76%
CookWare	308.34 min	73%	3 min	80%
DBTotal	24 min	75%	8.1 min	90%
Books	348 min	53%	3.85 min	93%
Hotel	382.4 min	60%	3.92 min	87%
Computers	392 min	67%	4.25 min	93%

**Table 10:** *P* value corresponding to classification accuracy and execution time.

Metric	*P* value (wrapper)	*P* value (CFS)
Classification accuracy	0.0008321	0.004
Execution time	0.000581	0.008
